# The public and patient involvement imperative in Ireland: Building on policy drivers

**DOI:** 10.3389/fpubh.2022.1038409

**Published:** 2022-11-10

**Authors:** Meghan Gilfoyle, Anne MacFarlane, Ailish Hannigan, Vikram Niranjan, Zoe Hughes, Jon Salsberg

**Affiliations:** ^1^Public and Patient Involvement Research Unit, School of Medicine, University of Limerick, Limerick, Ireland; ^2^Health Research Institute, University of Limerick, Limerick, Ireland; ^3^School of Public Health, Physiotherapy and Sports Science, University College Dublin, Dublin, Ireland; ^4^Care Alliance Ireland, Dublin, Ireland

**Keywords:** participatory health research, public and patient involvement (PPI), meaningful involvement, policy, co-design, health service research, methodological, community-based participatory research (CBPR)

## Abstract

What can we learn from the history of Public and Patient Involvement (PPI) in healthcare and research across global jurisdictions? Depending on region and context, the terminology and heritage of involvement in research vary. In this paper, we draw on global traditions to explore dominant themes and key considerations and critiques pertaining to PPI in order to inform a PPI culture shift in Ireland. We then describe the heritage of PPI in Ireland and present the case for combining methodological imperatives with policy drivers to support and encourage *meaningful* involvement. Specifically, we propose that PPI can be enriched by the theory and processes of participatory health research (PHR); and that implementation requires concurrent capacity building. We conclude with a call for Irish researchers (authors of this paper included) to consider the conceptual complexities and nuances of a participatory approach to build on the policy imperatives driving PPI and to contribute to the international evidence base and research culture. Specifically, we call for Irish health researchers and funders to consider and reflect on: (1) the rich literature of PHR as a resource for enacting meaningful PPI; (2) the roots and origins of varying participatory health research methods; (3) how community/patient groups can lead health research; and (4) co-learning and partnership synergy to create space for both academic and community expertise; and (5) the importance of using standardized reporting tools.

## Introduction

Evidence shows that involving patients and members of the public across crucial stages of research improves both process and outcomes and renders invaluable additional insights which could have otherwise been missed (1–3). The public and patients' contribution to the design, implementation, and evaluation of research leads to increased effectiveness, credibility, and often more cost-efficiency (4). Public and patient involvement (PPI) in health research thus addresses the modern imperative that high-quality research must bring real benefits for patients and other beneficiaries in their daily lives (5). Increasingly, research with PPI is becoming the encouraged norm in many jurisdictions (i.e., USA, UK, Canada, Australia).

Depending on region and context, the terminology and heritage of research involvement varies. A review by Boote et al. (6), exploring public involvement in health research between 1995 and 2009, emphasized that the UK, USA, Canada and Australia had the largest body of published work in this area. Further, a report published by the Australian Health Research Alliance in 2018, identified four leading agencies for promoting involvement, from the UK, USA, Canada and Australia (7). Thus, in this paper we draw on traditions from these countries when exploring dominant terms, traditions, and key considerations/critiques pertaining to collaborative research and practice (described in Additional File 1). As members of the Irish health research community, we are interested in exploring the multiple drivers for PPI and notable regional differences in the heritage of PPI.

In this paper, we critically reflect on the role of policy and argue that policy messaging can be enhanced if it is combined with clear messaging about the methodological gains of PPI. In doing so, we believe this will optimize the conditions for PPI to become the norm in practice. We describe the heritage of PPI in Ireland and present the case for combining methodological imperatives with policy ones to support and encourage the normalization of *meaningful* involvement. Drawing on the work by Cornwall (8, 9), when we say meaningful involvement, we mean that patients and members of the public have both the power and control to be equitably involved (as they see fit) in all levels of decision making and that *via* the participatory process, are facilitated to overcome both social and structural barriers to exercise such power. By normalization we mean that PPI is a routinised way of working that is integrated into stakeholders' daily practice (10).

## Drivers for PPI in international settings: An overview

Over the past decade, policy-driven initiatives in the USA and Canada have promoted greater *patient engagement*, currently the predominant term used in North America. The US Patient-Centered Outcomes Research Institute (PCORI), a health research funder formed under the Affordable Care Act (11, 12), has promoted a research culture that links funding to authentic stakeholder engagement, where stakeholders are communities, patients, or public and community organizations (13). As described by Woolf et al. (13), authentic stakeholder engagement is a term used to “characterize the involvement of all relevant stakeholders in all phases of research.” Similarly, Canada's Strategy for Patient-Oriented Research (SPOR) (14, 15) is a concerted policy drive to fund research that addresses patient-centered outcomes with the collaboration of patients and other members of the public. SPOR defines collaboration as “working in common cause with partners and key stakeholders on the development and implementation of the Strategy and on achieving its goals” (15). There are notable examples of community/patient drivers such as the need for patient centered outcomes (16, 17) spearheaded by organizations like the USA PCORI, and the right for patients to be involved in their own healthcare decision making (18). There are also new networks of academics and practitioners advocating for PPI capacity building [e.g., the North American Primary Care Research Group (NAPCRG), the International Collaboration for Participatory Health Research (ICPHR), and the Integrated Knowledge Translation Research Network (IKTRN)]. Further, there are examples of policy drivers from governmental departments and agencies including health research funders (19–21).

In Australia, collaborative research is commonly referred to as consumer-led research or consumer and community engagement/involvement (CCE). Examples of CCE as described by the National Health and Medical Research Council (NHMRC), include public consultation, representation on NHMRC committees, community and consumer advisory groups and on peer review panels (22). These examples stem from the NHMRC Act 1992, which depicts the statutory responsibility of the NHMRC “to raise the standard of individual and public health throughout Australia and foster the development of consistent health standards between various states and territories” (22). In line with this statutory responsibility, certain CCE engagement activities are mandated by the state (i.e., procedures and requirements for meeting the 2011 NHMRC standard for clinical practice guidelines) (22). These examples of involvement in health research are complemented by a policy foundation of involvement in health services (23, 24).

Comparatively, in the UK, the genesis of PPI is often framed as a response to “public demands for a greater voice in decisions about their services, and demands from politicians for greater efficiency, quality of services and effectiveness in the use of public funds” (25). The various PPI initiatives often reflected these demands, again as a policy imperative that became mandated by the governing authority at a given time. As Gibson et al. (25) discussed, “PPI is now more than ever embedded as an official ideology in legislation, and apparently official practice at all levels and in every aspect of policy” (25). PPI is thus situated as a key element in health and social care research in the UK, receiving strong policy support and active promotion through organizations such as INVOLVE and emphasized by funding bodies such as the National Institute for Health Research (20, 21).

Despite these ever-evolving policy traditions of PPI and the opportunities presented for involvement, there are challenges in these jurisdictions with PPI *in practice*. Sustained involvement is infrequently achieved (4) in part due to superficial, often tokenistic, engagement on the part of researchers. Taken simply as a policy imperative (i.e., do it because we say so), conflicting political values, ideologies and agendas of both researchers and public partners can impact the outcome of any involvement initiative (25). Further, as legislative policy provides guidance on PPI for commissioners of health services, such guidance is described as “open to interpretation” fostering varying approaches to the practice of PPI and, thus, varying outcomes (26). Indeed, it can be challenging to determine the outcomes of PPI when evaluations are based on initiatives that may not have effectively or meaningfully involved patients and the public at all (27). This leads to concerns about how to achieve genuine involvement that is not tokenistic, impacting the improvements in quality and efficiency (25). Specifically, Madden et al. (28), discuss that in this current context “PPI operates as an empty signifier, intermittently populated with whatever policy ideas of citizen engagement are *a la mode*.”

## Drivers for PPI in Ireland

Notwithstanding notable examples of internationally recognized meaningful PPI in the Irish context [i.e., (29, 30)] and important patient/community driven initiatives [e.g., (31, 32)], PPI is still in its formative days in Ireland as a normalized way of researching. We position the heritage, terminology and considerations for PPI in Ireland in comparison to other countries in Additional File 1. As in other countries, policies in Ireland have been in place for some time about service user involvement in health policy and service development. This includes the Health Service Executive National Strategy for Service User Involvement 2008–2013 (33), as well as Health Research Board (HRB) funding initiatives like the joint funding scheme with the Health Research Charities Ireland (HRCI, formally Medical Research Charities Group) (2006) or the Knowledge Exchange and Dissemination Scheme (2012) (34). However, PPI in health research remains relatively nascent. Arguably, it was not until 2014 that PPI became a *focal* priority explicitly discussed by funders and health researchers in Ireland (29, 30). That year, HRCI held its first ever Irish Health Research Forum to provide “a single Irish voice for research to improve health” with the focal theme of PPI (35). This forum was the first national health research discussion of “PPI as a priority” in Ireland (35). It was also in 2014 that HRB funding applications first included a question on PPI, but not as a mandatory assessment criterion (19). Specifically, most HRB funding calls ask researchers to explain how PPI will be incorporated in all stages of the research cycle, and if not why (36).

In 2016, the HRB Strategy 2016–2020 included its first explicit strategic commitment to “develop and promote PPI within the HRB and in HRB supported projects and programmes” (37). This included a new public review process, creating a panel of public reviewers who have contributed to the scoring of applications within at least seven HRB funding streams since 2018. Importantly, learning from other countries' experiences, both good and bad, the HRB recognized the need to *build capacity* to support PPI prior to mandating it in funding applications. In 2017, the HRB launched the “PPI Ignite Award,” a 3-year programme to build capacity and influence institutional research culture within Irish higher education institutions (38). In 2020 this transitioned into the 5-year “PPI Ignite Network,” expanding on the progress of the initial programme with more of a national rather than institutional focus (39) (see https://ppinetwork.ie). Moving forward, the HRB strategy 2021–2025 is “committed to ensuring that people remain at the very heart of everything we do” (37). PPI will be mandated by the HRB and will feature in the scoring of grant applications in the coming years. Thus, like other countries, policy drivers have played an important role in Ireland but, unlike other countries, the HRB's *approach has been incremental*, committing space and opportunity for building PPI knowledge and competencies within the health research community.

With regard to capacity building, as suggested by O'Shea et al. (40), Ireland can benefit from other countries' successes in relation to optimal approaches to PPI in health research (18, 41, 42). Ireland does not have to reinvent the wheel, e.g., initiatives like that of INVOLVE (43), have available resources on good practice and approaches to PPI in the UK, including a *Values and Principles Framework* (40). There may, of course, still be a role for national resources where there are gaps (44) or where adaptations are needed for the Irish context (45), but these represent advances or modifications to existing resources and foundations for good practice.

Accompanying these opportunities to learn from the successes of other countries, we must be mindful of challenges that may impede progress, and set us on a path of tokenism, if not fully considered. For instance, we must consider the limitations of approaching PPI *simply* as a policy imperative. If PPI is implemented *only* because it is a policy imperative, and *without capacity building* for it to be implemented meaningfully, it can reinforce existing power asymmetries between the academy and community. If, for example, the decisions about which community members are invited to participate in projects [the legitimate public, see Barnes et al. (46) vs. the usual suspects, see Beresford (47)] and if their role is pre-defined by academics in terms of how they should behave [what is sayable or doable by them in the research meetings, see Renedo and Martin (8, 48)], then the capacity for meaningful contributions is diminished. Further, an emphasis solely on policy mandates can obscure the methodological imperatives for, and benefits of, PPI and the growing evidence base about their positive impact on the generation and use of actionable knowledge from research.

To promote a PPI culture in Ireland, health researchers and funders should consider building on policy imperatives by looking beyond the “because we are told to,” message. We have the opportunity to reinforce the ethical and moral obligations for PPI, as well as recognizing the emergent evidence of methodological impact (18, 49, 50). Building on the considerations and critiques discussed above and described in Additional File 1, we suggest a way forward.

## The way forward: Participatory health research

As discussed by Gibson et al. (25) it is important to consider the emancipatory perspective and framework for PPI in health and social care to ensure that we are not harnessing a “PPI industry” fueled by imperatives at the system-level (such as government health policies), which can become more focused on efficiency and outputs than the experiences, needs and concerns of the public and patients (25). The moral, ethical, and methodological drivers for community and end-user involvement, discussed by Cargo and Mercer (25, 51), are reflected in the origins and practice of participatory health research (PHR).

PPI can be enriched by the theory and processes of PHR, defined as research undertaken in collaboration with those affected by the issue being studied, for the purposes of taking action or effecting change (52). PHR has a rich tradition of literature, resources and evidence about the rationale for and value of partnerships. Promoting multiple ways of knowing, while highlighting relational and reflective knowledge as well as transformative learning, PHR strives for broad impact (53). There are two historical traditions that describe the origin of PHR: the Northern tradition, striving for societal change through action research (54) and the Southern tradition, striving for social justice and emancipation through self-determination (55). Lewin's action research (the origin of modern implementation models) (54) speaks most directly to the knowledge utilization driver, while Freire's work in critical pedagogy resonates most closely with the drivers of social justice and self-determination (55).

For example, in the USA, for more than four decades, communities have mobilized to broaden the involvement of people and organizations in research to address community-level problems related to health and social issues (56). The recognition and understanding of the impact of the community voice in effectively and efficiently achieving challenging health objectives, led to increased investment in community partnerships and participation initiatives by USA agencies (57–59). As mentioned earlier, in the USA, PCORI has been a major champion of this shift in expectations (11, 12). For instance, PCORI has followed through/developed its policy mandate for patient engagement by promoting a research culture that links funding to the authentic stakeholder engagement characteristic of participatory health research (13).

A growing body of evidence has accumulated recognizing the methodological and impact benefits from PHR's value base. For example, a review by Jagosh et al. (1), discussed PHR's benefits from a methodological perspective, such as generating greater recruitment capacity, as well as impacts, such as stakeholder competency and capacity and sustained partnerships. As described in a position paper by the ICPHR (60), “impact through PHR is embedded in a dialogical process of critical reflection in and on action (60),” through its collaborative and emancipatory roots exploring the needs and issues pertinent to the community. Through reflexive practice, co-learning and action, transformative knowledge is entrenched in the process in doing PHR (60). There are also a variety of tools and techniques in the PHR literature that can be used to support partnerships (i.e., sharing the decision-making, data generation and co-analysis) with diverse stakeholders, for example, participatory learning and action (61, 62). This highlights that it is incumbent on researchers to think critically and creatively about the methods they use to involve stakeholders in research.

There are gaps in knowledge about PHR internationally. For example, Hannigan (63) argues for the need for more direct involvement of partners in quantitative data analysis and statistical modeling. Patients and the public have been described as the missing stakeholder group in the modeling process and the benefits of participatory approaches to modeling are increasingly being recognized (64, 65). Quantitative data are “not just numbers, they are numbers with a context,” and a key strength of PHR is better understanding context (51).

While the HRB in Ireland does not expressly employ a PHR framework, it does emphasize some important processes that resonate with its principles (such as involving people early in the research process, or later in dissemination planning). This is similar to Canada's SPOR, which scores grant applications on patient or community involvement at different research stages (14, 15). An example within the Irish context, where aligning with PHR has explicitly shaped PPI in research, is that of the HRB-funded PPI Ignite programme at the University of Limerick. As described earlier, the purpose of the 2017 PPI Ignite Award was to support universities to build capacity for involving patients and members of the public in health research. The University of Limerick (UL) took the decision to approach PPI by drawing on the rich tradition of PHR, adopting its participatory principles and practices with a multi-sector audience. Specifically, *PPI Ignite@UL* (66) has co-developed with health sector, community and patient organizations who directly co-governed the project and partnered in creating and deploying training and development activities. These partners also contributed to evaluating the programme's products and outcomes. For more information on how the PHR approach was important for capacity building see Additional File 2. This work continues to be developed in the PPI Ignite Network (described earlier), alongside additional successful national initiatives such as the PPI Festival (see https://ppinetwork.ie/festival/).

## Discussion

### Need for more consistent PPI reporting

There has been a significant lack of reporting on involvement within this field, and subsequently a lack of consistency with reporting when it does occur (63, 67–73). Capturing and documenting wider forms of impact remains underrepresented in published accounts of research evidence (60). This is problematic for many reasons, but arguably, at the forefront of this issue is the lack of available, or non-fragmented evidence to assess impact, impeding “our collective understanding of what works, for whom, why, and in what context” (69). As discussed by Staniszewska et al. (69), many of the papers published “provide little information on how members were involved and the results of this involvement.” Staley (73), posits that this problem is 2-fold: (1) there is a problem for assessing impact; and (2) there is a lack of structure and guidance on involvement in peer-reviewed journals.

This issue of reporting, however, is not due to the lack of tools, frameworks, guidelines, and critical appraisal checklists available for public and patient involvement in research, as demonstrated in a systematic review by Greenhalgh et al. (71). This review (71) sought to identify, synthesize and critically examine the published frameworks available for use, further identifying if they had been actually used and why. The most recent and arguably most accepted reporting framework is the new “Guidance for Reporting Involvement of Patients and the Public 2” (GRIPP2), which precedes its earlier version GRIPP (69).

However, are researchers using these frameworks? This question is explored in the second objective of the systematic review by Greenhalgh et al. (71). For the reporting guidelines available at the time of the review, the study had not identified any papers describing the use of the framework, beyond those who developed it (69).

### Call for Irish health researchers

Issues identified by Staley (73) such as, inefficient, standardized, and inadequate reporting, continue to plague this field. These issues need to be addressed to achieve a better understanding of how certain variables/processes/constructs within the partnership process are impacting health outcomes.

We now call for Irish health researchers (authors included) and funders to consider and reflect on: (1) the rich literature of PHR as a resource for enacting meaningful PPI; (2) the roots and origins of varying participatory health research methods; (3) how community/patient groups can lead health research; and (4) co-learning and partnership synergy to create space for both academic and community expertise; and (5) the importance of using standardized reporting tools. Specifically, Irish researchers could use these lessons to ensure a PPI trajectory that moves away from tokenism and a checklist approach to partnerships by also using moral, ethical, and methodological drivers for PPI in health research. By approaching this incrementally and allowing researchers and their partners to gain comfort and competency in PPI, the HRB is wisely avoiding some of the pitfalls experienced in other jurisdictions. PHR provides theoretical and methodological resources to *enact* key values that support and create meaningful and sustainable partnerships that, in turn, improves the *quality* of PPI with scope for positive outcomes on the *process* and *outcomes* of partnered research.

## Data availability statement

The original contributions presented in the study are included in the article/Supplementary material, further inquiries can be directed to the corresponding author/s.

## Author contributions

MG conceptualized, wrote the first draft of the paper, and applied edits based on feedback from other listed authors. JS, AM, and AH provided thorough revisions and feedback to all drafts. AM also drafted Additional File 2. VN contributed to background and literature synthesis and writing. ZH helped to conceptualize the paper and provided revisions and feedback of drafts. All authors made substantial contribution to the conception, writing and reviewing of the work, and have approved the submitted version of this work.

## Funding

This work was supported by the GEMS-10 scholarship from the University of Limerick (UL) (Ireland) and a scholarship from the Integrated Knowledge Translation Research Network (Canada: CIHR Foundation Grant; FDN #143237). This work is partially supported by the HRB/IRC PPI Ignite@UL network grant (Grant # HRB PPI-2017-009).

## Conflict of interest

The authors declare that the research was conducted in the absence of any commercial or financial relationships that could be construed as a potential conflict of interest.

## Publisher's note

All claims expressed in this article are solely those of the authors and do not necessarily represent those of their affiliated organizations, or those of the publisher, the editors and the reviewers. Any product that may be evaluated in this article, or claim that may be made by its manufacturer, is not guaranteed or endorsed by the publisher.

## Abbreviations

PPI: public and patient involvement
PCORI: patient-centered outcomes research institute
SPOR: strategy for patient-oriented research
CCE: consumer and community engagement
NHMRC: national health and medical research council
HRB: health research board
HRCI: health research charities Ireland
PHR: participatory health research
UL: University of Limerick.
