# Supplementary material for: The public and patient involvement imperative in Ireland: Building on policy drivers
Source: Front Public Health. 2022 Nov 10;10:1038409. doi: 10.3389/fpubh.2022.1038409 (PMC9684639; doi:10.3389/fpubh.2022.1038409)
Supplement: Supplementary file 1 [file Table_1.PDF]

Additional File 1 – Heritage and terminology of collaborative practice

Terminology/Definition	Jurisdiction	Heritage/Roots	Considerations
Public and Patient Involvement<br>"Research being carried out '**with**' or '**by**' members of the public rather than '**to**', '**about**' or '**for**' them." (1)	**United Kingdom**	• **1974:** the first formal structures to represent the public's interest in the NHS were created, called the Community Health Councils (CHCs). (2)<br>• **1991:** Launch of the NHS Research and Development Strategy, the UK recognised public involvement in health research as policy. (3)<br>• **1996:** the UK Department of Health established INVOLVE promoting its policy of public involvement to health researchers. (4)<br>• **2003:** CHCs were abolished and fragmented into several organisations, including Overview and Scrutiny Committees (OSCs—the remit of which was extended to cover healthcare), Patient Advice and Liaison Service (PALS), Independent Complaints Advocacy Service (ICAS) and Patient and Public Involvement Forums (PPIfs). (2) These forums aimed at improving engagement in NHS organizations, but criticized in that their members didn't adequately or accurately reflect views of local communities.(5)<br>• **2008:** PPIfs abolished and replaced by Local Involvement Networks (LINKs), with the aim of improving engagement with local community and voluntary organisations with a local area. (5)<br>• **2010:** Elected government unveils plan for the future of PPI within the NHS, wherein NHS Trusts have duties to ***prove*** patient involvement. (5)<br>• PPI is now embedded as an ideology in legislation, and official practice at all levels and in every aspect of policy. (5)<br>• In the United Kingdom, PPI has mostly taken the form of indirect involvement, where information and opinions are gathered from service users by health professionals and managerial staff to inform service delivery and development; but the clinicians and managers make the final decisions. (6) The patient and the NHS have always been central elements of health policy in the UK. (6) In recent years, there have been NHS structural changes to ensure public involvement at	• As a policy imperative, conflicting political values, ideologies and agendas present at the time impact the outcome of any involvement initiative. This leads to concerns regarding genuine involvement that isn't tokenistic, impacting the improvements in quality and efficiency. (5)

	**Ireland**	every level, by increasing local capacity for participation. (7-9)<br>• **2014**: PPI became a *focal* priority explicitly discussed by funders and health researchers in Ireland.<br>○ In 2014, when Health Research Charities Ireland (HRCI) held its first ever Irish Health Research Forum to provide “a single Irish voice for research to improve health,” (10) with the focal theme of PPI. This forum was the first national health research discussion of ‘PPI as a priority’ in Ireland. (11)<br>○ In 2014 that HRB funding applications first included a question on PPI, but not as a mandatory assessment criterion. (12) Specifically, most HRB funding calls ask researchers to explain how PPI will be incorporated in all stages of the research cycle, and if not why. (13)<br>• **2016:** HRB implemented the “the HRB Strategy 2016–2020” including its first explicit strategic commitment to “develop and promote PPI within the HRB and in HRB supported projects and programmes.” (14) This included a new public review process, creating a panel of public reviewers who have contributed to the scoring of applications within at least seven HRB funding streams since 2018.<br>• **2017**: the HRB launched the “PPI Ignite Award”, a 3-year programme to build capacity and influence institutional research culture within Irish higher education institutions. (15, 16)<br>• **2020:** the HRB launches the 5-year “National PPI Network”, expanding on the progress of the initial programme with more of a national – rather than institutional focus.(17)<br>• **2021-2025** the HRB strategy is “committed to ensuring that people remain at the very heart of everything we do.”(14) PPI will be mandated by the HRB and will feature in the scoring of grant applications in the coming years.	• As a PPI approach to health research is deployed and evolves in Ireland, it is possible to use these lessons to ensure a PPI trajectory that moves away from tokenism and PPI checklists by also using moral, ethical, and methodological drivers for PPI in health research. By approaching this incrementally and allowing researchers and their partners to gain comfort and competency in PPI, the HRB is avoiding some of the pitfalls experienced in other jurisdictions.
Patient Engagement<br>“occur[ing] when patients meaningfully and actively collaborate in the governance, priority setting, and conduct of research, as well as in	**United States and Canada**	• Rooted in the civil rights movement in the 1960’s, leading towards more patient empowerment. (20)<br>• Women’s health movement of the 1970s and 1980s. (21)	

summarizing, distributing, sharing, and applying its resulting knowledge." (18, 19)		• AIDS movement of the 1980s and 1990s, "nothing about us without us." (21)	
Participatory Research<br>"Systematic inquiry, with the collaboration of those affected by the issue being studies, for the purposes of education and taking action or effecting change." (22)	**United States**<br><br>**Canada**	• Largest body of literature and longest lineage among the approaches. (23)<br>• Origins in social action research and an emancipatory philosophy. (23)<br>• Two historical traditions for this approach:<br>1) Northern Tradition; Kurt Lewin, a German social psychologist, coined the term 'action research' in the 1940's. Lewin aimed to promote cyclical practices of planning, action and investigating the results of action, while involving community members and workers in workers in collaborative manner, striving for societal change. (24, 25) This tradition originated in the USA, South America and the UK. (25)<br>2) Southern Tradition; described as 'emancipatory research', which was inspired by Paulo Freire, an exiled Brazilian philosopher, in the 1970s in South America. (24, 25) Aims to "promote practices and strategies that engage oppressed, marginalised, disadvantaged or disempowered communities within a dominant society, primarily Latin America, Africa and Asia, to affect policy Change." (25)	PPI can be enriched by the theory and processes of participatory health research. PHR has a rich tradition of literature, resources, and evidence about the rationale for and value of partnerships. Promoting multiple ways of knowing, while highlighting relational and reflective knowledge as well as transformative learning, PHR strives for broad impact. (26)
Engaged Scholarship<br>Two levels:<br>1. Transformation of academia and reinvigorating academic systems and structure. (25)<br><br>2. Encouraging researchers to reconnect with social issues through engagements. (25)	**USA**	• Concept was first coined by the educator Ernest Boyer in the 1990's. (25)<br>• Boyer suggested the imperativeness of public engagement in research by conducting research that focuses on societal needs rather than individual or institutional needs. (25)<br>• Intent was "education for democracy, civic responsibility/engagement and public scholarship facilitated by university researchers." (25)	
Consumer and Community Engagement/Involvement/Leadership<br>***"***Consumer [and community] engagement addresses the need to increase citizen's awareness of, and involvement in, in health-related decisions such as the: prioritizing research and allocation of funding and	**Australia**	**1992:** a report of a national workshop on consumer participation in public health research was published which strongly emphasised PPI. (31)<br>Publish the National Mental Health Policy, (32) with direction on the consumer involvement in mental health services. (30)<br>**1997:** Publish the National Standards for Mental Health Services (33) discussing consumer involvement in mental	

the design and delivery of interventions aimed at enhancing public health and reducing health inequality. The process can involve different aspects or elements, such as ‘patient involvement, participation, collaboration, education, and empowerment(27)’.” (28)<br><br>“At the micro level, it means being actively engaged in clinical service planning and treatment decisions. In other words, it is about being a partner in the clinical process rather than being merely compliant with the clinical decisions made by experts. At the macro level, it means contributing to decisions about the way services operate, including planning and reform processes. Here the consumer or carer is acting not just in relation to personal treatment but to broader processes that impact on larger groups of consumers and carers. In other words, it is a representative role.” (29, 30)		health services, (30) with a focus on outcomes for consumers and carers. (29)<br>**1998:** Publish the Second National Mental Health Plan(34) with further direction on the consumer involvement in mental health services (30), and partnership development as a key priority in service delivery and change. (29)<br>**1999:** The Wills report made recommendations on how the public should be involved in health research conducted in the country. (35)<br>**2000:** The National Health and Medical Research Council (NHMRC) agree to fund the Consumers’ Health Forum of Australia Inc proposal to develop a statement on community and consumer participation in health and medical research. (36)<br>**2002:** The Statement on Consumer and Community and Participation in Health and Medical research released, which aims to provide a “reference point for involving consumers and the community in research and in communication about the role, benefits and results of research...” (36)<br>**2007: “**The Australian Conduct of Research (2007), the primary guidance for institutions and research in responsible research practices, states: ‘Appropriate consumer involvement in research should be encouraged and facilitated by research institutions and researchers.’ In addition, NHMRC encourages researchers to consider the benefits of actively engaging consumers in their proposed research, when they apply to NHMRC for research funding.” (37)<br>**2016:** The NHMRC and the CHF release a statement (replacing that from 2002) titled “the Statement on Consumer and Community Involvement in Health and Medical Research”, with “the purpose to guide research institutions, researchers, consumers and community members in the active involvement of consumers and community members in all aspects of health and medical research.” (37)<br>**2020:** In consultation with members of the Community and Consumer Advisory Group, the NHMRC releases a suite of resources related to consumer and community involvement in, and expectations of, health and medical research. (38)	

References

1. INVOLVE. Public involvement in research: Values and principles framework. Eastleigh, UK: Nihr Involve. (2015).
2. Department of Health. Patient and public involvement in health: The evidence for policy implementation: Department of Health; (2004).
3. Barron K. Patient and public involvement in the NHS: third report of session 2006-07, Vol. 2: Oral and written evidence: The Stationery Office; (2007).
4. Gray-Burrows KA, Willis TA, Foy R, Rathfelder M, Bland P, Chin A, et al. Role of patient and public involvement in implementation research: a consensus study. BMJ Quality & Safety. (2018);27(10):858-64.
5. Gibson A, Britten N, Lynch J. Theoretical directions for an emancipatory concept of patient and public involvement. Health:. (2012);16(5):531-47.
6. Tritter JQ. Public and patient participation in health care and health policy in the United Kingdom. Health expectations: an international journal of public participation in health care and health policy. (2011);14(2):220.
7. Tritter JQ. Revolution or evolution: the challenges of conceptualizing patient and public involvement in a consumerist world. Health Expectations. (2009);12(3):275-87.
8. Rowe R, Shepherd M. Public participation in the new NHS: no closer to citizen control? Social Policy & Administration. (2002);36(3):275-90.
9. Hughes D, Mullen C, Vincent-Jones P. Choice vs. voice? PPI policies and the re-positioning of the state in England and Wales. Health Expectations. (2009);12(3):237-50.
10. Health Research Charities Ireland (HRCI). Irish Health Research Forum (IHRF) [Available from: https://hrci.ie/about-us/our-work/about-ihrf/.
11. Irish Health Research Forum (IHRF). Document on: Public and Patient Involvement (PPI) in Research. 2015.
12. Hendrick S, Tedstone D, Cody A. Evaluation of HRB public review pilot–summary. Health Research Board. (2017).
13. Health Research Board (HRB). Secondary Data Analysis Projects (SDAP) 2021 - Call guidance document Online(2021) [Available from: https://www.hrb.ie/fileadmin/2._Plugin_related_files/Funding_schemes/SDAP_2021_Guidance_Notes.pdf.
14. Health Research Board (HRB). Strategy 2021–2025. (2021). Available from: https://www.hrb.ie/fileadmin/2._Plugin_related_files/Publications/2021_publications/2021_Corp/Strategy_2021_2025_Health_research_making_an_impact.pdf.
15. Board HR. Research. Evidence. Action. Strategy 2016-2020. (2016). Available from: https://www.hrb.ie/fileadmin/publications_files/HRB_Strategy_2016-2020.pdf.
16. Health Research Board (HRB). PPI Ignite Awards 2017 - Supporting Public and Patient Involvement in Research. [Internal Document]. In press 2016.

17. Health Research Board (HRB). National PPI Network (2021) - Supporting Public and Patient Involvement in Research: Health Research Board; (2020) [Available from: https://www.hrb.ie/fileadmin/2._Plugin_related_files/Funding_schemes/National_PPI_Network_Guidance_Notes_Final.pdf.
18. Manafo E, Petermann L, Mason-Lai P, Vandall-Walker V. Patient engagement in Canada: a scoping review of the 'how'and 'what'of patient engagement in health research. Health research policy and systems. (2018);16(1):1-11.
19. Research CIoH. Strategy for patient-oriented research putting patients first patient Engagament framework. (2014).
20. Archambault PM, McGavin C, Dainty KN, McLeod SL, Vaillancourt C, Lee JS, et al. Recommendations for patient engagement in patient-oriented emergency medicine research. CJEM. (2018);20(3):435-42.
21. Stroud IE. Patient engagement: a powerful tool with a powerful history (2014) [Available from: https://thrivingwithlungcancer.com/2014/01/29/patient-engagement-a-powerful-tool-with-a-powerful-history/.
22. Green LW, Canada RSo, Research BCfHP, editors. Study of participatory research in health promotion: Review and recommendations for the development of participatory research in health promotion in Canada1995: Royal Society of Canada; (Year) Published.
23. Nguyen T, Graham ID, Mrklas KJ, Bowen S, Cargo M, Estabrooks CA, et al. How does integrated knowledge translation (IKT) compare to other collaborative research approaches to generating and translating knowledge? Learning from experts in the field. Health research policy and systems. (2020);18(1):1-20.
24. Wallerstein N, Duran B, Oetzel JG, Minkler M. Community-based participatory research for health: advancing social and health equity: John Wiley & Sons; (2017).
25. Nguyen T, Graham ID, Mrklas KJ, Bowen S, Cargo M, Estabrooks CA, et al. How does integrated knowledge translation (IKT) compare to other collaborative research approaches to generating and translating knowledge? Learning from experts in the field. Health Research Policy and Systems. (2020);18(1):35.
26. International Collaboration for Participatory Health Research (ICPHR). Position paper 1: What is participatory health research? : ICPHR Berlin; 2013.
27. Goss C, Mosconi P, Renzi C, Deledda G. Participation of patients and citizens in healthcare decisions in Italy. Zeitschrift für Evidenz, Fortbildung und Qualität im Gesundheitswesen. (2011);105(4):277-82.
28. Sarrami-Foroushani P, Travaglia J, Debono D, Braithwaite J. Implementing strategies in consumer and community engagement in health care: results of a large-scale, scoping meta-review. BMC Health Serv Res. (2014);14:402-.
29. Lloyd C, King R. Consumer and carer participation in mental health services. Australasian Psychiatry. (2003);11(2):180-4.
30. Gordon S. The role of the consumer in the leadership and management of mental health services. Australasian Psychiatry. (2005);13(4):362-5.
31. Watt A. Health and Medical Research in Australia: Observatory on Health Research Systems. Santa Monica, CA: RAND Corporation; (2008).

32. Australian Health Ministers C. National mental health policy / Australian Health Ministers. Canberra: Australian Govt. Pub. Service; (1992).
33. National standards for mental health services. Australian Health Ministers' Advisory Council. National Working Group on Mental Health P, editor. Canberra: Australian Govt. Pub Service; (1997).
34. Second national mental health plan / Australian Health Ministers. Australia. Department of H, Family Services. Mental Health B, National Mental Health S, editors. Canberra: Commonwealth Department of Health and Family Services; (1998).
35. Wills P. The virtuous cycle, working together for health and medical research : summary / Health and Medical Research Strategic Review. Australia H, Medical Research Strategic R, editors. Canberra: Dept. of Health and Aged Care; (1998).
36. National Health & Medical Research Council; Consumers' Health Forum of Australia. Statement on Consumer and Community
Participation in Health and Medical Research. 2001.
37. Consumers Health Forum of Australia. Statement on Consumer and Community involvement in Health and Medical Research. National Health and Medical Research Council; 2016.
38. The National Health and Medical Research Council. Consumer and Community Engagement Online: Australian Government, National Health and Medical Research Council; [Available from: https://www.nhmrc.gov.au/about-us/consumer-and-community-engagement.
