# Supplementary material for: The public and patient involvement imperative in Ireland: Building on policy drivers
Source: Front Public Health. 2022 Nov 10;10:1038409. doi: 10.3389/fpubh.2022.1038409 (PMC9684639; doi:10.3389/fpubh.2022.1038409)
Supplement: Supplementary file 2 [file Table_2.PDF]

Additional File 2

A Participatory Health Research approach to capacity building in the University of Limerick, Ireland

PPI Ignite @UL 2017-2020 was designed based on some key principles of PHR.
The *proposal was co-designed* with the stakeholders that the capacity building was intended for: community and patient groups, healthcare providers and academics in the region. This led to an agreed programme with three work packages for training, networking and policy change.

Stakeholders had *representation on the project's governance structures* and there was shared decision-making throughout the programme about the budget and development of each work package.

During the course of the project, stakeholders:

1. *co-designed and evaluated the training* in PHR leading to six workshops that are now embedded in the University's Human Resources Training and Development Programme (introduction to PHR; ethics and PHR; finding research partners; collaborative grant development; PHR for quantitative and qualitative studies, collaboration data analysis). Some stakeholders were involved as *co-facilitators* in the delivery of these workshops
2. *co-designed networking events* to allow community and patient groups, healthcare providers and academics in the region to meet and discuss their research interests. This led to some new partnerships to inform collaborative design of new research proposals
3. *co-designed plans to improve the policies and procedures* in the university to support, for example, funding arrangements between the university and community organisations. This work was challenging but has led to new understanding in the Finance and Research Office about the mechanisms that need to change.
